# Potency of Tokishakuyakusan in treating preeclampsia: Drug repositioning method by *in vitro* screening of the Kampo library

**DOI:** 10.1371/journal.pone.0244684

**Published:** 2020-12-30

**Authors:** Kazunobu Yagi, Kazuya Mimura, Takuji Tomimatsu, Tatsuya Matsuyama, Yoko Kawanishi, Aiko Kakigano, Hitomi Nakamura, Masayuki Endo, Tadashi Kimura

**Affiliations:** Department of Obstetrics and Gynecology, Osaka University Graduate School of Medicine, Suita, Japan; University of Mississippi Medical Center, UNITED STATES

## Abstract

**Introduction:**

Preeclampsia therapy has not been established, except for the termination of pregnancy. The aim of this study was to identify a potential therapeutic agent from traditional Japanese medicine (Kampo) using the drug repositioning method.

**Materials and methods:**

We screened a library of 74 Kampo to identify potential drugs for the treatment of preeclampsia. We investigated the angiogenic effects of these drugs using human umbilical vein endothelial cells (HUVECs). Enzyme-linked immunosorbent assays were performed to measure the levels of placental growth factor (PlGF) in conditioned media treated with 100 μg/mL of each drug. We assessed whether the screened drugs affected cell viability. We performed tube formation assays to evaluate the angiogenic effects of PlGF-inducing drugs. PlGF was measured after administering 10, 50, 100, and 200 μg/mL of the candidate drug in the dose correlation experiment, and at 1, 2, 3, 6, 12, and 24 h in the time course experiment. We also performed tube formation assays with the candidate drug and 100 ng/mL of soluble fms-like tyrosine kinase 1 (sFlt1). PlGF production by the candidate drug was measured in trophoblastic cells (BeWo and HTR-8/SVneo). The Mann-Whitney U test or one-way analyses of variance followed by the Newman-Keuls post-hoc test were performed. P–values < 0.05 were considered significant.

**Results:**

Of the 7 drugs that induced PlGF, Tokishakuyakusan (TS), Shoseiryuto, and Shofusan did not reduce cell viability. TS significantly facilitated tube formation (P = 0.017). TS administration increased PlGF expression in a dose- and time-dependent manner. TS significantly improved tube formation, which was inhibited by sFlt1 (P = 0.033). TS also increased PlGF production in BeWo (P = 0.001) but not HTR-8/SVneo cells (P = 0.33).

**Conclusions:**

By using the drug repositioning method in the *in vitro* screening of the Kampo library, we identified that TS may have a therapeutic potential for preeclampsia. Its newly found mechanisms involve the increase in PlGF production, and improvement of the antiangiogenic state.

## Introduction

Preeclampsia is a major complication of pregnancy detected in 2–8% of pregnant women [1]. Its treatment is one of the main challenges in the field of perinatology, and has a significant impact on the health of the mother and her offspring [2, 3]. To date, apart from termination of pregnancy, there is no treatment for preeclampsia. The 2-stage model is the most accepted explanation for its pathogenesis [4]. Stage 1 involves a decrease in uteroplacental perfusion secondary to poor placentation. This results in excessive secretion of soluble fms-like tyrosine kinase 1 (sFlt1) from the ischemic placenta [5–7]. Stage 2 occurs later in the disease process and involves maternal systemic endothelial dysfunction [4]. It is believed that sFlt1 binds to angiogenic factors (placental growth factor [PlGF] and vascular endothelial growth factor [VEGF]) to attenuate their action, leading to endothelial cell damage. If we want to based on the 2-stage model, a therapeutic drug that can increase PlGF and inhibit the systemic antiangiogenic state could be a potential treatment for preeclampsia.

However, the development of new drugs is challenging in that it is time-consuming and costly [8]. The greatest concern arises, especially in pregnant women, due to potential teratogenicity in the fetus [9]. A proposed solution to this problem is drug repositioning. The drug repositioning method involves the investigation of existing drugs for new therapeutic indications [10]. This method can enable an easy investigation of the effects of the drugs at minimal toxicity levels since the toxicity of the latter is already known [10–13]. This strategy is especially beneficial for pregnant women, where existing drugs are preferred over novel drugs for safety purposes. From a study involving an *in vitro* screening of 528 drugs approved in Japan, we previously reported that vardenafil increased PlGF production and had angiogenic effects [14]. Vardenafil is widely used as a phosphodiesterase-5 (PDE5) inhibitor. A commonly known mechanism of PDE5 inhibitors is the release of nitric oxide (NO) which causes vasodilation by preventing the degradation of cyclic guanosine monophosphate. We discovered that vardenafil had a novel mechanism: it increases PlGF release from endothelial cells. PDE5 inhibitors are possible therapeutics agents. Research on the use of these agents for preeclampsia is ongoing. However, sildenafil (a PDE5 inhibitor) was found to increase neonatal death [15], making its clinical use in pregnant women stringent.

Traditional Japanese herbal medicine, known as Kampo, originating from Chinese medicine was introduced in Japan between the 5^th^ and 6^th^ centuries. Kampo is often considered a safe alternative to synthetically manufactured drugs [16]. An increasing number of patients and medical practitioners—not only in Asia, but also in Western countries—use Kampo as complementary or alternative therapy [17]. Since its approval by the Ministry of Health, Labor, and Welfare in 1976, Kampo has been widely employed in Japan. Kampo medicine has been used in Japanese pregnant women for a long time and is generally considered safe for the fetus. In this study, we screened the approved Kampo drug library to identify any drug with angiogenic effects that could potentially be used to treat preeclampsia.

## Materials and methods

### Kampo library

We used a library of 74 Kampo drugs that are generally used in Japan. These Kampo drugs were obtained from Tsumura & Co. (Tokyo, Japan). The compositions and ratios of the crude drugs included in these Kampo drugs are shown in Table 1. The Kampo drugs were dissolved in 10 mg/mL concentrations of ultra-pure water at 50°C for 1 hour. Using the techniques employed in previous reports, we purified the drugs by low-speed centrifugation, and then filtered it using a 0.22 *μ*m filter. The final drug was stored at -30°C before use [18, 19].

**Table 1:** Library of 74 approved Kampo drugs screened for therapeutic effects in preeclampsia, and the quantity of crude drugs (g) per daily dose.

	Kampo drug	Product No.	Crude drug
1	Kakkonto	1	Kakkon (4), Kanzo (2), Keihi (2), Shakuyaku (2), Shokyo (2), Taiso (3), Mao (3)
2	Kakkontokasenkyushini	2	Kakkon (4), Kanzo (2), Keihi (2), Shakuyaku (2), Shokyo (1), Shini(2), Senkyu (2), Taiso (3), Mao (3)
3	Otsujito	3	Ogon (3), Kanzo (2), Saiko (5), Shoma (1), Daio (0.5), Toki (6)
4	Anchusan	5	Uikyo (1.5), Engosaku (3), Kanzo (1), Keihi (4), Shukusha (1), Borei (3), Ryokyo (0.5)
5	Hachimijiogan	7	Keihi (1), Sanshuyu (3), Sanyaku (3), Jio (6), Takusha (3), Bukuryo (3), Bushi (0.5), Botanpi (2.5)
6	Daisaikoto	8	Ogon (3), Kijitsu (2), Saiko (6), Shakuyaku (3), Shokyo (1), Daio (1), Taiso (3), Hange (4)
7	Saikokeishito	10	Ogon (2), Kanzo (2), Keihi (2), Saiko (5), Shakuyaku (2), Shokyo (1), Taiso (2), Ninjin (2), Hange (4)
8	Saikokeishikankyoto	11	Ogon (3), Karokon (3), Kankyo (2), Kanzo (2), Keihi (3), Saiko (6), Borei (3)
9	Saikokaryukotsuboreito	12	Ogon (2.5), Keihi (3), Saiko (5), Shokyo (1), Taiso (2.5), Ninjin (2.5), Hange (4), Bukuryo (3), Borei (2.5), Ryukotsu (0.5)
10	Orengedokuto	15	Ogon (3), Obaku (1.5), Oren (2), Sanshishi (2)
11	Keishikajutsubuto	18	Kanzo (2), Keihi (4), Shakuyaku (4), Shokyo (1), Sojutsu (4), Taiso (4), Bushi (0.5)
12	Shoseiryuto	19	Kankyo (3), Kanzo (3), Keihi (3), Gomishi (3), Saishin (3), Shakuyaku (3), Hange (6), Mao (3)
13	Shofusan	22	Kanzo (1), Kujin (1), Keigai (1), Goboshi (2), Goma (1.5), Jio (3), Sekko (3), Zentai (1), Sojutsu (2), Chimo (1.5), Toki (3), Bofu (2), Mokutsu (2)
14	Tokishakuyakusan	23	Shakuyaku (4), Senkyu (3), Sojutsu (4), Takusha (4), Toki (3), Bukuryo (4)
15	Kamishoyosan	24	Kanzo (1.5), Saiko (3), Sanshishi (2), Shakuyaku (3), Shokyo (1), Sojutsu (3), Toki (3), Hakka (1), Bukuryo (3), Botanpi (2)
16	Keishibukuryogan	25	Keihi (3), Shakuyaku (3), Tonin (3), Bukuryo (3), Botanpi (3)
17	Keishikaryukotsuboreito	26	Kanzo (2), Keihi (4), Shakuyaku (4), Shokyo (1.5), Taiso (4), Borei (3), Ryukotsu (3)
18	Shinbuto	30	Shakuyaku (3), Shokyo (1.5), Sojutsu (3), Bukuryo (4), Bushi (0.5)
19	Shigyakusan	35	Kanzo (1.5), Kijitsu (2), Saiko (5), Shakuyaku (4)
20	Tokishigyakukagoshuyushokyoto	38	Kanzo (2), Keihi (3), Goshuyu (2), Saishin (2), Shakuyaku (3), Shokyo (1), Taiso (5), Toki (3), Mokutsu (3)
21	Hochuekkito	41	Ogi (4), Kanzo (1.5), Saiko (2), Shokyo (0.5), Shoma (1), Sojutsu (4), Taiso (2), Chimpi (2), Toki (3), Ninjin (4)
22	Keishito	45	Kanzo (2), Keihi (4), Shakuyaku (4), Shokyo (1.5), Taiso (4)
23	Shichimotsukokato	46	Ogi (3), Obaku (2), Jio (3), Shakuyaku (4), Senkyu (3), Chotoko (3), Toki (4)
24	Chotosan	47	Kanzo (1), Kikuka (2), Shokyo (1), Sekko (5), Chotoko (3), Chimpi (3), Ninjin (2), Bakumondo (3), Hange (3), Bukuryo (3), Bofu (2)
25	Juzentaihoto	48	Ogi (3), Kanzo (1.5), Keihi (3), Jio (3), Shakuyaku (3), Senkyu (3), Sojutsu (3), Toki (3), Ninjin (3), Bukuryo (3)
26	Keigairengyoto	50	Ogon (1.5), Obaku (1.5), Oren (1.5), Kanzo (1), Kikyo (1.5), Kijitsu (1.5), Keigai (1.5), Saiko (1.5), Sanshishi (1.5), Jio (1.5), Shakuyaku (1.5), Senkyu (1.5), Toki (1.5), Hakka (1.5), Byakushi (1.5), Bofu (1.5), Rengyo (1.5)
27	Junchoto	51	Ogon (2), Kanzo (1.5), Kijitsu (2), Kyonin (2), koboku (2), Jio (6), Daio (2), Toki (3), Tonin (2), Mashinin (2)
28	Yokuininto	52	Kanzo (2), Keihi (3), Shakuyaku (3), Sojutsu (4), Toki (4), Mao (4), Yokuinin (8)
29	Sokeikakketsuto	53	Ireisen (1.5), Kanzo (1), kyoukatu (1.5), goshitu (1.5), Jio (2), Shakuyaku (2.5), Shokyo (0.5), Senkyu (2), Sojutsu (2), Chimpi (1.5), Toki (2), Tonin (2), Byakushi (1), Bukuryo (2), Boi (1.5), Bofu (1.5), Ryutan (1.5)
30	Yokukansan	54	Kanzo (1.5), Saiko (2), Senkyu (3), Sojutsu (4), Chotoko (3), Toki (3), Bukuryo (4)
31	Gorinsan	56	Ogon (3), Kasseki (3), Kanzo (3), Sanshishi (2), Jio (3), Shakuyaku (2), Shazenshi (3), Takusha (3), Toki (3), Bukuryo (6), Mokutsu (3)
32	Unseiin	57	Ogon (1.5), Obaku (1.5), Oren (1.5), Sanshishi (1.5), Jio (3), Shakuyaku (3), Senkyu (3), Toki (3)
33	Seijobofuto	58	Ogon (2.5), Oren (1), Kanzo (1), Kikyo (2.5), Kijitsu (1), Keigai (1), Sanshishi (2.5), Senkyu (2.5), Hakka (1), Hamabofu (2.5), Byakushi (2.5), Rengyo (2.5)
34	Jizusoippo	59	Kanzo (1), Keigai (1), Koka (1), Senkyu (3), Sojutsu (3), Daio (0.5), Nindo (2), Bofu (2), Rengyo (3)
35	Keishikashakuyakuto	60	Kanzo (2), Keihi (4), Shakuyaku (6), Shokyo (1), Taiso (4)
36	Bofutsushosan	62	Ogon (2), Kasseki (3), Kanzo (2), Kikyo (2), Keigai (1.2), Sanshishi (12), Shakuyaku (1.2), Shokyo (0.3), Sekko (2), Senkyu (1.2), Daio (1.5), Toki (1.2), Hakka (1.2), Byakujyutsu (2), Bosho (0.1), Bofu (1.2), Mao (1.2), Rengyo (1.2)
37	Goshakusan	63	Kaznou (1), Kikyo (1), Kijitsu (1), Keihi (1), Koboku (1), Shakuyaku (1), Shokyo (1), Senkyu (1), Sojutsu (3), Taiso (1), Chimpi (2), Toki (2), Hange (2), Byakushi (1), Bukuryo (2), Mao (1)
38	Shakanzoto	64	Akyo (2), Keihi (3), Jio (6), Shakanzo (3), Shokyo (1), Taiso (3), Ninjin (3), Bakumondo (6), Mashinin (3)
39	Kihito	65	Ogi (3), Onji (2), Kanzo (1), Sansonin (3), Shokyo (1), Taiso (2), Toki (2), Ninjin (3), Byakujyutsu (3), Bukuryo (3), Mokko (1), Ryuganniku (3)
40	Jinsoin	66	Kakkon (2), Kanzo (1), Kikyo (2), Kijitsu (1), Shokyo (0.5), Zenko (2), Soyo (1), Taiso (1.5), Chimpi (2), Ninjin (1.5), Hange (3), Bukuryo (3)
41	Nyoshinsan	67	Ogon (2), Oren (1), Kanzo (1), Keihi (2), Kobushi (3), Senkyu (3), Sojutsu (3), Choji (1), Toki (3), Ninjin (2), Binroji (2), Mokko (1)
42	Shakuyakukanzoto	68	Kanzo (6), Shakuyaku (6)
43	Shimotsuto	71	Jio (3), Shakuyaku (3), Senkyu (3), Toki (3)
44	Ryutanshakanto	76	Ogon (3), Kanzo (1), Sanshishi (1), Jio (5), Shazenshi (3), Takusha (3), Toki (5), Mokutsu (5), Ryutan (1)
45	Kyukikyogaito	77	Akyo (3), Gaiyo (3), Kanzo (3), Jio (5), Shakuyaku (4), Senkyu (3), Toki (4)
46	Saikoseikanto	80	Ogon (1.5), Obaku (1.5), Oren (1.5), Karokon (1.5), Kanzo (1.5), Kikyo (1.5), Goboshi (1.5), Saiko (2), Sanshishi (1.5), Jio (1.5), Shakuyaku (1.5), Senkyu (1.5), Toki (1.5), Hakka (1.5), Rengyo (1.5)
47	Yokukansankachimpihange	83	Kanzo (1.5), Saiko (2), Senkyu (3), Sojutsu (4), Chotoko (3), Chimpi (3), Toki (3), Hange (5), Bukuryo (4)
48	Tokiinshi	86	Ogi (1.5), Kashu (2), Kanzo (1), Keigai (1.5), Jio (4), Shitsurishi (3), Shakuyaku (3), Senkyu (3), Toki (5), Bofu (3)
49	Jidabokuippo	89	Kanzo (1.5), Keihi (3), Senkyu (3), Senkotsu (3), Daio (1), Choji (1), Bokusoku (3)
50	Seihaito	90	Ogon (2), Kanzo (1), Kikyo (2), Kyonin (2), Gomishi (1), Sanshishi (2), Shokyo (1), Sohakuhi (2), Taiso (2), Chikujyo (2), Chimpi (2), Tenmondo (2), Toki (3), Baimo (2), Bakumondo (3), Bukuryo (3)
51	Jiinshihoto	92	Kanzo (1), Kobushi (3), Saiko (3), Jikoppi (3), Shakuyaku (3), Chimo (3), Chimpi (3), Toki (3), Baimo (2), Bakumondo (3), Hakka (1), Byakujyutsu (3), Bukuryo (3)
52	Jiinkokato	93	Obaku (1.5), Kanzo (1.5), Jio (2.5), Shakuyaku (2.5), Sojutsu (3), Chimo (1.5), Chimpi (2.5), Tenmondo (0.5), Toki (2.5), Bakumondo (2.5)
53	Daibofuto	97	Ogi (3), Kankyo (1), Kanzo (1.5), Kyokatsu (1.5), Goshitsu (1.5), Jio (3), Shakuyaku (3), Senkyu (2), Sojutsu (3), Taiso (1.5), Toki (3), Tochu (3), Ninjin (1.5), Bushi (1), Bofu (3)
54	Ogikenchuto	98	Ogi (4), Kanzo (2), Keihi (4), Shakuyaku (6), Shokyo (1), Taiso (4)
55	Shokenchuto	99	Kanzo (2), Keihi (4), Shakuyaku (6), Shokyo (1), Taiso (4)
56	Shomakakkonto	101	Kakkon (5), Kanzo (1.5), Shakuyaku (3), Shokyo (0.5), Shoma (2)
57	Tokito	102	Ogi (1.5), Kankyo (1.5), Kanzo (1), Keihi (3), Koboku (3), Sansho (1.5), Shakuyaku (3), Toki (5), Ninjin (3), Hange (5)
58	Sansoninto	103	Kanzo (1), Sansonin (10), Senkyu (3), Chimo (3), Bukuryo (5)
59	Tsudosan	105	Kanzo (2), Kijitsu (3), Koka (2), Koboku (2), Soboku (2), Daio (3), Chimpi (2), Toki (3), Bosho (1.8), Mokutsu (2)
60	Unkeito	106	Akyo (2), Kanzo (2), Keihi (2), Goshuyu (1), Shakuyaku (2), Shokyo (1), Senkyu (2), Toki (3), Ninjin (2), Bakumondo (4), Hange (4), Botanpi (2)
61	Ninjinyoeito	108	Ogi (1.5), Onji (2), Kanzo (1), Keihi (2.5), Gomishi (1), Jio (4), Shakuyaku (2), Chimpi (2), Toki (4), Ninjin (3), Byakujyutsu (4), Bukuryo (4)
62	Choreitogoshimotsuto	112	Akyo (3), Kasseki (3), Jio (3), Shakuyaku (3), Senkyu (3), Takusha (3), Chorei (3), Toki (3), Bukuryo (3)
63	Saireito	114	Ogon (3), Kanzo (2), Keihi (2), Saiko (7), Shokyo (1), Sojutsu (3), Taiso (3), Takusha (5), Chorei (3), Ninjin (3), Hange (5), Bukuryo (3)
64	Ireito	115	Kanzo (1), Keihi (2), Koboku (2.5), Shokyo (1.5), Sojutsu (2.5), Taiso (1.5), Takusha (2.5), Chorei (2.5), Chimpi (2.5), Byakujyutsu (2.5), Bukuryo (2.5)
65	Orento	120	Oren (3), Kankyo (3), Kanzo (3), Keihi (3), Taiso (3), Ninjin (3), Hange (6)
66	Hainosankyuto	122	Kanzo (3), Kikyo (4), Kijitsu (3), Shakuyaku (3), Shokyo81), Taiso (3)
67	Tokikenchuto	123	Kanzo (2), Keihi (4), Shakuyaku (5), Shokyo (1), Taiso (4), Toki (4)
68	Senkyuchachosan	124	Kanzo (1.5), Kyokatsu (2), Keigai (2), Kobushi (4), Senkyu (3), Chayo (1.5), Hakka (2), Byakushi (2), Bofu (2)
69	Keishibukuryogankayokuinin	125	Keihi (4), Shakuyaku (4), Tonin (4), Bukuryo (4), Botanpi (4), Yokuinin (10)
70	Mashiningan	126	Kijitsu (2), Kyonin (2), Koboku (2), Shakuyaku (2), Daio (4), Mashinin (5)
71	Daijokito	133	Kijitsu (3), Koboku (5), Daio (2), Bosho (1.3)
72	Keishikashakuyakudaioto	134	Kanzo (2), Keihi (4), Shakuyaku (6), Shokyo (1), Daio (2), Taiso (4)
73	Seishoekkito	136	Ogi (3), Obaku (1), Kanzo (1), Gomishi (1), Sojutsu (3.5), Chimpi (3), Toki (3), Ninjin (3.5), Bakumondo (3.5)
74	Kamikihito	137	Ogi (3), Onji (2), Kanzo (1), Saiko (3), Sanshishi (2), Sansonin (3), Shokyo (1), Sojutsu (3), Taiso (2), Toki (2), Ninjin (3), Bukuryo (3), Mokko (1), Ryuganniku (3)

### Isolation and culture of primary human endothelial cells

After approval by Osaka University Ethics Committee, we isolated and cultured human umbilical vein endothelial cells (HUVECs) from the umbilical cords [20]. Donors were recruited from pregnant women who delivered at 37–41 weeks of gestation between January 2019 and September 2020 at the Osaka University Hospital, Osaka, Japan. We included only women who were more than 20 years old and had singleton pregnancies with normal blood pressure and appropriate for gestational age fetuses. We excluded women who had stillbirth, multiple gestations, fetuses with congenital malformations or chromosomal abnormalities, fetal growth restriction, preeclampsia and maternal medical complications. Written informed consent was obtained from all participants. The cultures were maintained at 37°C in a humidified atmosphere containing 5% CO_2_. In this experiment, the cells underwent 3 to 5 rounds of subcultures.

### Culture of trophoblastic cells (HTR-8/SVneo and BeWo cells)

HTR-8/SVneo cells (subsequently referred to as HTR-8 cells) were obtained from ATCC^®^ (CRL3271™) and cultured in RPMI-1640 culture medium (Nacalai Tesque, Japan). BeWo cells were obtained from Riken Cell Bank (Ibaraki, Japan) and cultured in Dulbecco’s Modified Eagle Medium/Ham F-12 culture medium (Nacalai Tesque, Japan). The medium was supplemented with 10% fetal bovine serum (FBS), penicillin (100 IU/mL), and streptomycin (100 mg/mL).

### Enzyme-linked immunosorbent assay

Human PlGF was measured using enzyme-linked immunosorbent assay (ELISA) kits (SPG00, R&D Systems, Minneapolis, MN, USA) [20]. We seeded HUVECs in 96-well plates at a density of 1×10^4^ cells/well. After 24 hours, the Kampo drugs were put into the subconfluent cells with an M199 medium containing 0.1% FBS. Controls were administered with M199 containing 0.1% FBS and vehicle (ultra-pure water). The PlGF concentrations in the conditioned media were measured. During the screening process, PlGF production was measured 24 h after administering 100 μg/mL of each drugs [14, 21]. During the first screening, two independent experiments were performed in a single assay. During the second screening, PlGF ELISA was repeated for drugs that produced a PlGF level more than 120% above the control in the first stage. Experiments were performed in triplicate. For the dose correlation experiment, the PlGF level was measured 24 h after the administration of 10, 50, 100, and 200 μg/mL of each candidate Kampo drug in triplicate. For the time course experiment, the PlGF level was measured at 1, 2, 3, 6, 12, and 24 h after incubation with 100 μg/mL of the candidate drug in triplicate. ELISA was also performed in HTR-8 and BeWo cells cultured at subconfluence in a 96 well-plate. As described above, PlGF concentrations in the conditioned media after 24 h of treatment were measured in triplicate.

### Cell viability assay

HUVECs were cultured in M199 containing 0.1% FBS (control) and one candidate Kampo drug at a concentration of 100 μg/mL for 24 h. Controls were cultured in M199 containing 0.1% FBS and vehicle (ultra-pure water). We performed a 3-(4,5-dimethylthiazol-2-yl)-5-(3-carboxymethoxyphenyl)2-(4-sulfophenyl)-2H-tetrazolium inner salt (MTS) assay using the CellTiter 96 AQueous One Solution Cell Proliferation Assay (Promega, Madison, Wisconsin) as previously described [20]. Cell Counting Kit-8 (CCK-8) (Dojindo, Kumamoto, Japan) using WST-8 [2-(2-methoxy-4-nitrophenyl)-3-(4-nitrophenyl)-5-(2,4-disulfophenyl)-2H-tetrazolium, monosodium salt] was also used to measure cell viability according to the manufacturer's protocol. In summary, HUVECs were plated in 96-well plates (at concentrations of 5×10^3^ cells/well) and 10 μL CCK-8 reagent was added into each well. The cells were incubated for 1 h. Cell proliferation was monitored at a wavelength of 450 nm. The CCK-8 assay was also performed in HTR-8 and BeWo cells as described above. Each exposure experiment was performed in triplicate.

### Tube formation assay

We performed a tube formation assay [20] to assess angiogenesis of endothelial cells [22]. The surfaces of 96-well plates were coated with growth factor-reduced Matrigel (BD Biosciences, Bedford, Massachusetts) according to the manufacturer’s instructions. HUVECs were starved in serum-free M199 for 8 h. Thereafter, the serum-starved cells were harvested into the Matrigel-coated wells (2×10^4^ cells per well) and treated with one of the screened drugs with or without sFlt1 (100 ng/mL) in M199 containing 0.1% FBS. Controls were treated with M199 containing 0.1% FBS and vehicle (ultra-pure water). After 8 h, the formation of microvascular tubes was observed using an inverted phase contrast microscope at 4x magnification. We measured the total tube length using the ImageJ imaging software (National Institutes of Health, Bethesda, Maryland). Each experiment was performed in triplicate.

### Statistical analysis

All statistical analyses were performed using the software JMP version 14.0 (SAS Institute, Cary, NC, USA). Mann-Whitney U test or one-way analysis of variance followed by the Newman-Keuls post-hoc test was used for statistical comparisons between groups. P-values < 0.05 were considered statistically significant.

## Results

**Fig 1:**
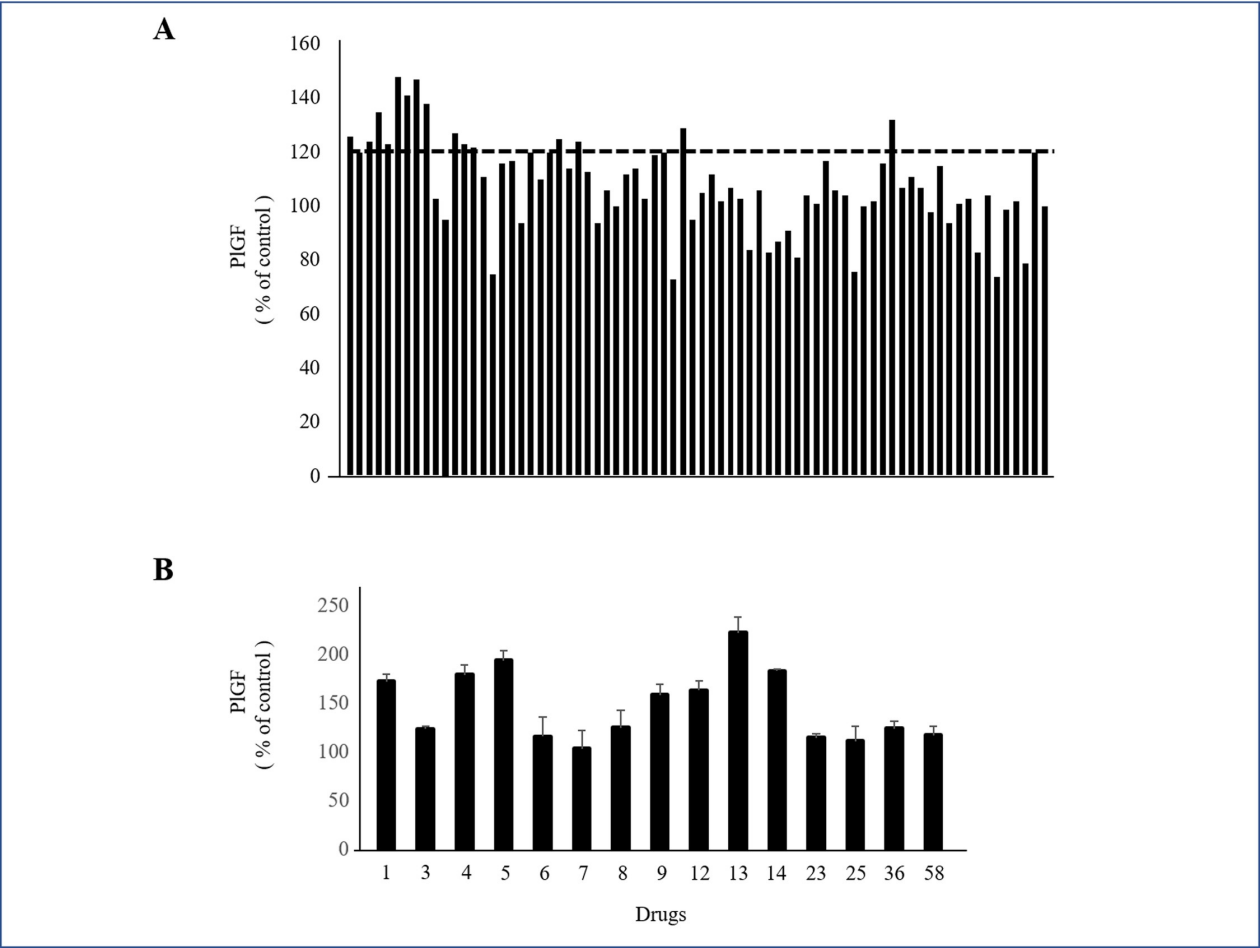
Production of PlGF from HUVECs treated with a library of Kampo drugs. **A**: First screening. PlGF concentrations produced by 74 drugs. **B**: Second screening. PlGF concentrations produced by 15 drugs increased PlGF by more than 120 above the control in the first screening. 1: Kakkonto; 3: Otsujito; 4: Anchusan; 5: Hachimijiogan; 6: Daisaikoto; 7: Saikokeishito; 8: Saikokeishikankyoto; 9: Saikokaryukotsuboreito; 12: Shoseiryuto; 13: Shofusan 14: Tokishakuyakusan 23: Shichimotsukokato; 25: Juzentaihoto; 36: Bofutsushosan; 58: Sansoninto. PlGF, placental growth factor; HUVECs, human umbilical vein endothelial cells.

**Fig 2:**
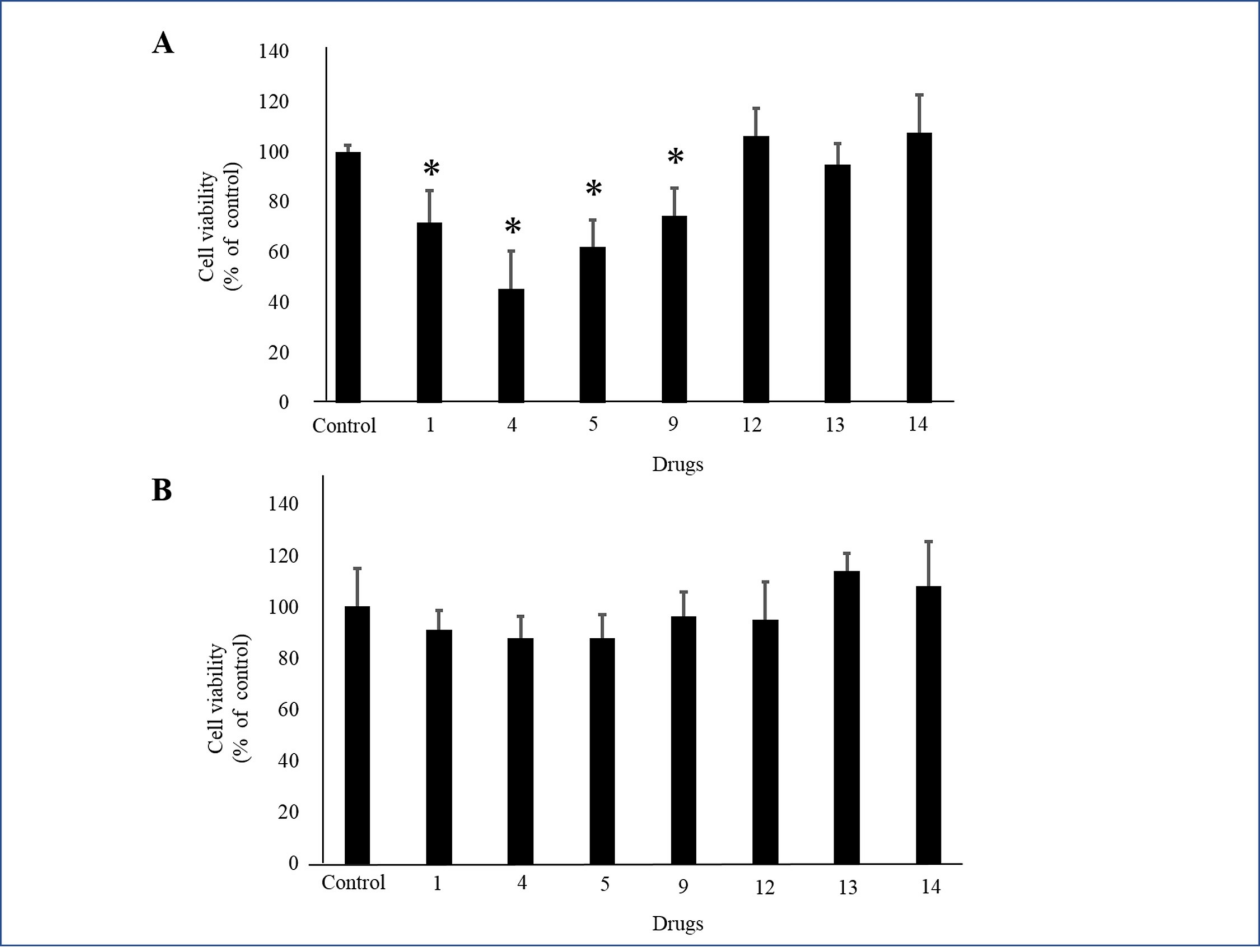
Effect of selected Kampo drugs on the viability of HUVECs. **A**: The MTS assay with one of 7 selected drugs. **B**: The CCK-8 assay with one of 7 selected drugs. * indicates P < 0.05. 1: Kakkonto; 4: Anchusan; 5: Hachimijiogan; 9: Saikokaryukotsuboreito; 12: Shoseiryuto; 13: Shofusan 14: Tokishakuyakusan. HUVECs, human umbilical vein endothelial cells; MTS, 3-(4,5-dimethylthiazol-2-yl)-5-(3-carboxymethoxyphenyl)2-(4-sulfophenyl)-2H-tetrazolium inner salt; CCK, Cell Counting Kit.

**Fig 3:**
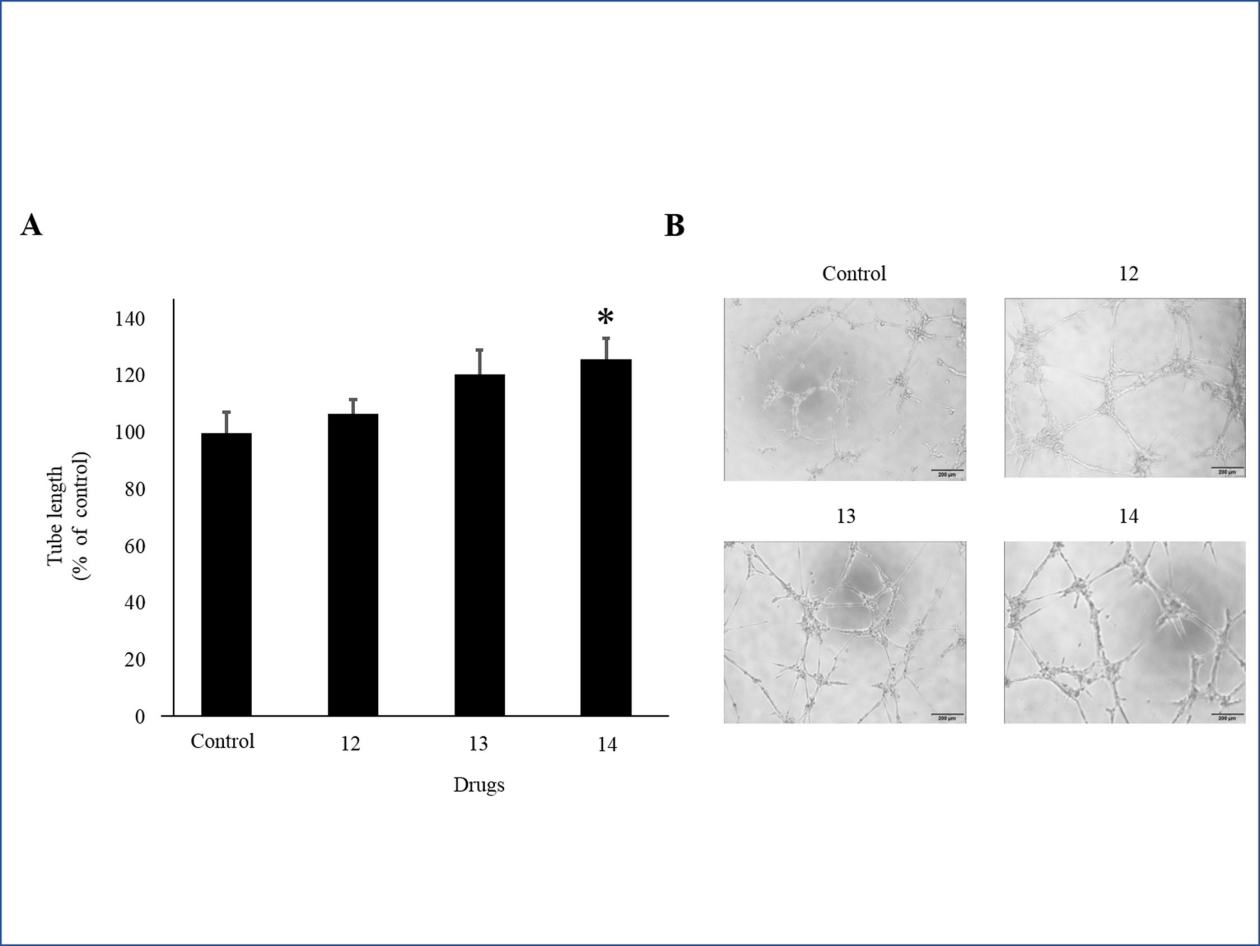
Effect of selected Kampo drugs on microvascular tube formation in HUVECs. **A:** Quantification of total tube length. **B:** Representative images. * indicates P < 0.05. 12: Shoseiryuto; 13: Shofusan; 14: Tokishakuyakusan. HUVECs, human umbilical vein endothelial cells.

**Fig 4:**
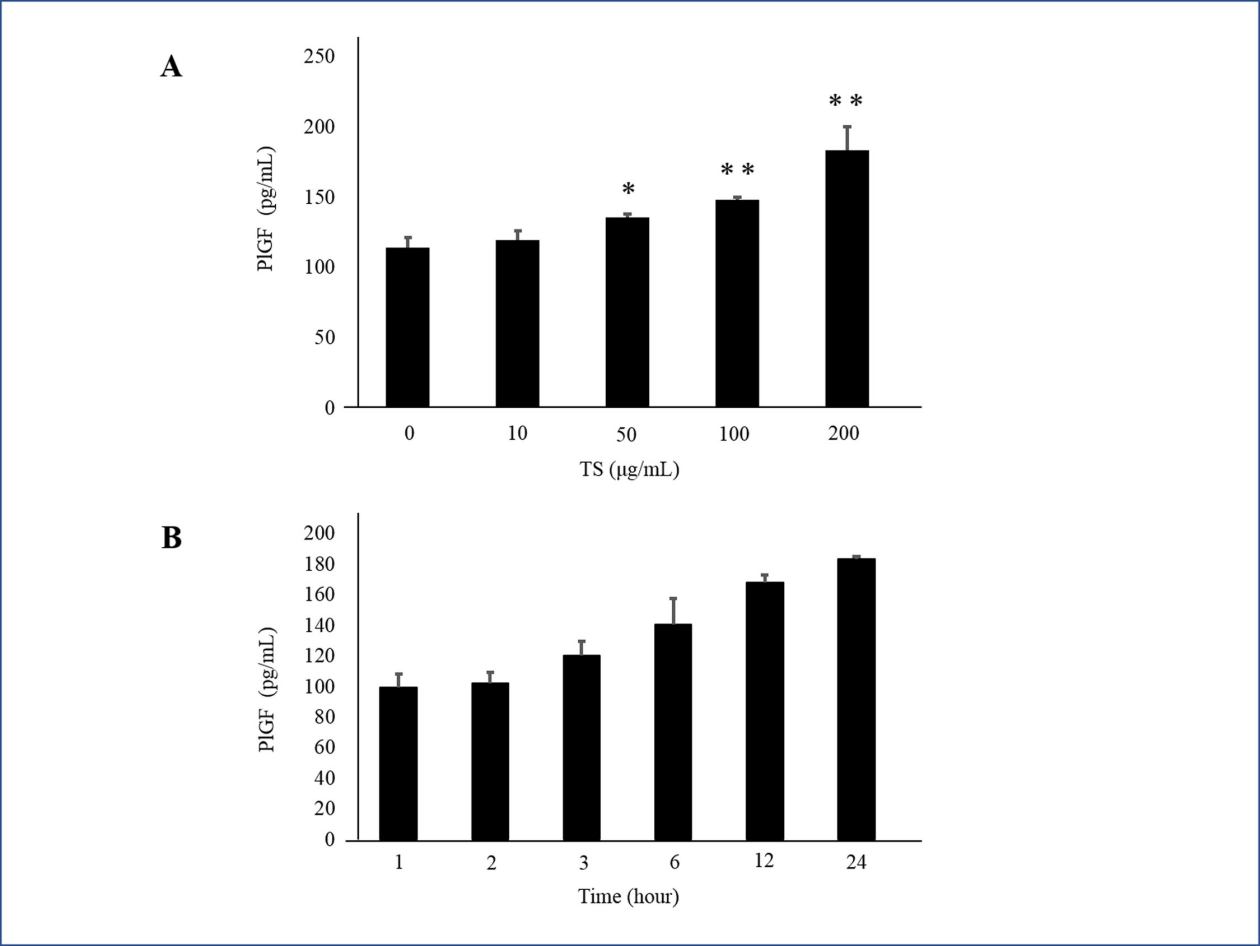
Effect of TS on PlGF expression in HUVECs. **A:** Dose correlation experiment. **B:** Time course experiment. * indicates P <0.05; ** indicates P <0.01. TS, Tokishakuyakusan; PlGF, placental growth factor; HUVECs, human umbilical vein endothelial cells.

**Fig 5:**
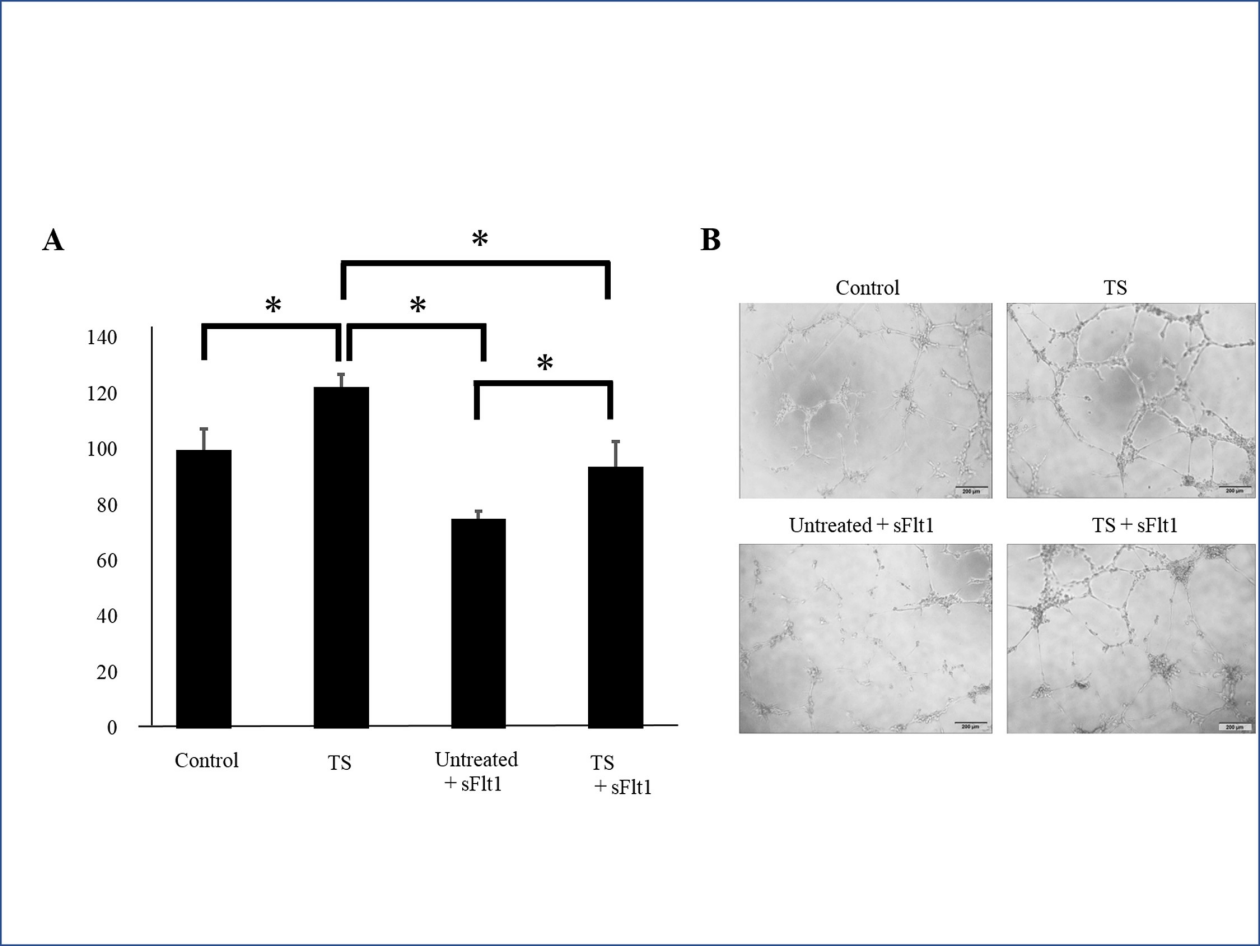
Effect of TS on tube formation of HUVECs with antiangiogenic factors. **A:** Quantification of total tube length. **B:** Representative images. * indicates P <0.05. TS, Tokishakuyakusan; HUVECs, human umbilical vein endothelial cells; sFlt1, soluble fms-like tyrosine kinase 1.

**Fig 6:**
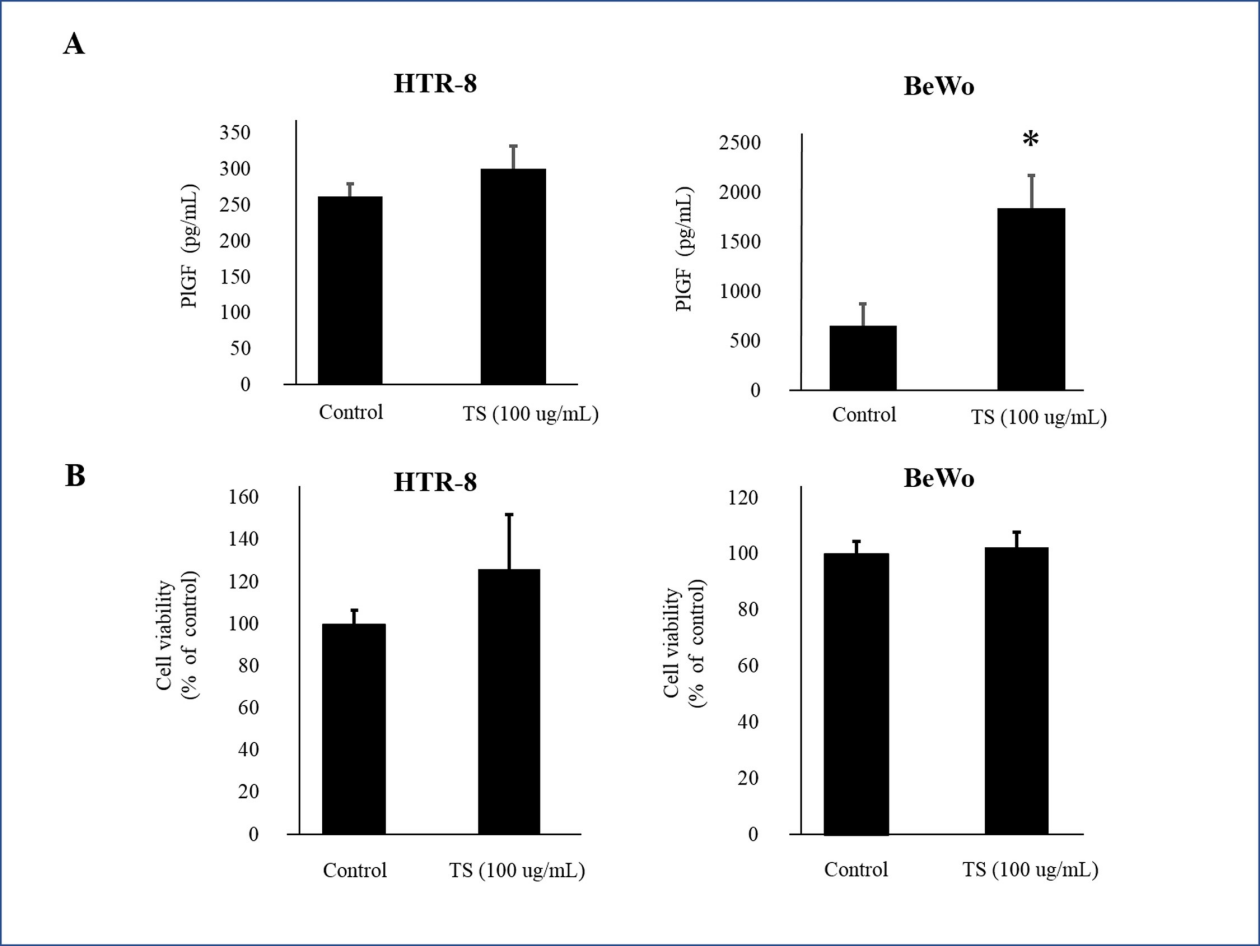
Effect of TS on PlGF expression and the cell viability of trophoblastic cells. **A:** PlGF concentrations produced by TS from HTR-8/SVneo and BeWo cells. **B:** The CCK-8 assay with TS in HTR-8/SVneo and BeWo cells. * indicates P <0.05. TS, Tokishakuyakusan; PlGF, placental growth factor; HTR-8, HTR-8/SVneo cells; BeWo, BeWo cells.

After the first screening process, 15 drugs produced PlGF levels more than 120% above the control (Fig 1A). PlGF ELISA was repeated on each of these 15 drugs (Fig 1B). We selected 7 drugs that produced the highest levels of PlGF: (1) Kakkonto, (4) Anchusan, (5) Hachimijiogan, (9) Saikokaryukotsuboreito, (12) Shoseiryuto, (13) Shofusan, and (14) Tokishakuyakusan (TS). In MTS assays, cell viability did not significantly decrease after administration of Shoseiryuto, Shofusan, and TS in comparison with controls (P = 0.76, P = 0.20 and P = 0.69, respectively) (Fig 2A). In CCK-8 assays, cell viability was not different between the 7 Kampo drugs and controls (Fig 2B). In the next step, we observed that the endothelial cells were associated with each other and formed microtubes after treatment with the aforementioned three drugs. TS significantly extended the total tube length when compared to the control treatment (P = 0.017) (Fig 3A). The other two drugs, however, did not extend tube length (P = 0.82 and P = 0.21, respectively) (Fig 3A). Fig 3B shows representative images. As shown in Fig 4A, TS at various concentrations (50, 100, and 200 μg/mL) after 24 h of incubation significantly stimulated PlGF production from HUVECs in a dose-dependent manner (P = 0.046, P = 0.01 and P = 0.02, respectively). A time course experiment of PlGF induction was also performed after treatment with 100 μg/mL of TS. We observed that PlGF levels increased with time until 24 h of incubation (Fig 4B). Co-administration of sFlt1 significantly impaired the microvascular tube formation when compared with the control (P = 0.003) (Fig 5A). Coincubation with TS significantly improved tube formation (P = 0.033) (Fig 5A). Fig 5B shows representative images. TS also significantly stimulated PlGF production from BeWo cells (P = 0.001) but not HTR-8 cells (P = 0.33) (Fig 6A). TS did not affect cell viability both in BeWo (P = 0.75) and HTR-8 cells (P = 0.31) during the CCK-8 assays (Fig 6B).

## Discussion

This was the first study to identify a candidate drug for treating preeclampsia by screening a library of Kampo drugs. This screening revealed a newly discovered mechanism of action of TS: it increases PlGF production and has angiogenic effects. In our study, many drugs failed to produce more PlGF than the controls. This result indicated the extreme difficulty of identifying drugs that could increase PlGF expression without doing drug screening. Drug repositioning is an efficient method to identify new drugs (in this case, with mechanisms against preeclampsia) that cannot be identified by conventional methods. Few reports have described the drug repositioning approach in preeclampsia treatment. In 2014, Rana et al. reported to have screened a library of 502 natural compounds using human placental cell lines and found that ouabain inhibited placental sFlt1 production [23]. However, our study was the only one (to our knowledge) that screened for drugs that increase the level of PIGF in vascular endothelial cells. We were able to assess the angiogenic effect of Kampo drugs for the first time.

In the present study, we showed that several Kampo drugs, including TS, increased PlGF production but only TS had angiogenic effects. The crude drugs commonly contained in three or more of the seven Kampo drugs that increased PlGF were Kanzo, Keihi, Shakuyaku and Bukuryo. Of these, TS contained Shakuyaku and Bukuryo. Of the seven Kampo drugs, Senkyu was the only crude drug contained in TS only. From these results, it seems insufficient to conclude on the existence of crude drugs that are key agents responsible for the increase in PlGF. Some crude drugs alone may increase PlGF, but synergistic effects have been reported to be important in Kampo medicine [24]. Therefore, the interaction of the crude drugs may increase PlGF secretion and produce angiogenic effects. Some Kampo medicines containing Koka, Goshitsu, Daio, and Kijitsu can cause uterine contractions; therefore, they are carefully administered during pregnancy. However, TS does not contain these ingredients. TS is also used for the treatment of preterm labor since it has tocolytic effects [25]. TS has been found to be non-teratogenic in an animal experiment conducted in rats [26]. In addition, there has been no report of teratology even when administered in humans during early pregnancy [27]. In our study, TS did not affect the cell viability. To investigate cell viability, MTS and CCK-8 assays were performed and several Kampo drugs reduced cell viability in the MTS assay but not the CCK-8 assay. The difference in the results from the two assays may be due to the low cytotoxicity of the reagents in the CCK-8 assay. In general, the detection sensitivity of CCK-8 is higher than that of other tetrazolium salts, including the MTS assay [28]. However, TS was not cytotoxic in both assays and may be safe to use.

Some researchers have demonstrated the ameliorative effects of TS on preeclampsia, fetal growth restriction, and recurrent pregnancy loss [29, 30]. It has been reported that TS regulates the immunity of invariant natural killer T cells during early pregnancy (in mice), and thus reduces the rate of pregnancy loss [31]. The antihypertensive effects of TS have also been reported [29]. However, there are no previous reports examining TS-induced PlGF expression. So far, pravastatin, nicotine, and vardenafil have been reported as drugs that increase the secretion of PlGF from vascular endothelial cells [14, 20, 32]. However, nicotine reduced PlGF levels in trophoblastic cells [33]. Although it is unknown whether TS has a higher capacity for PlGF production than these drugs, it was possible to show that it has an angiogenic effect in the tube formation assay. In addition, the fact that the angiogenic effect was also observed in the presence of sFlt1 indicated that the latter might have a protective effect on the vascular endothelium at stage 2 of the onset mechanism of preeclampsia. Considering the treatment of preeclampsia, it is important as TS has an angiogenic effect. This is because, apart from causing vasoconstriction, preeclampsia has a complex pathophysiology. Unlike many other antihypertensive drugs, TS does not lower blood pressure in normotensive pregnant rats [29]. This suggests that TS might be used more safely during pregnancy. Therefore, TS may be clinically used to treat preeclampsia due to its mechanism of increasing PlGF and enhancing angiogenic effects. In our study, TS also increased PlGF secretion in BeWo cells. So far, sulfasalazine has been reported to increase PlGF in trohoblastic cells. However, sulfasalazine reduced PlGF levels in vascular endothelial cells [34]. To the best of our knowledge, only TS has been found to increase PlGF levels both in vascular endothelial cells and in trohoblastic cells. This result enhances the potential of TS as a therapeutic agent for preeclampsia, because the main place of secretion of PlGF is the placenta. However, TS did not increase PlGF production in HTR-8 cells, which are the 1st trimester trohoblastic cells. Paradoxically, PlGF is reported to increase sFlt1 secretion from the developing placenta (stage 1), and may contribute to the onset of preeclampsia [35]. Therefore, it may be advantageous to use TS safely during the 1st trimester. The free radical scavenging activity of TS involves an antioxidant effect and may play a role in its antihypertensive action [36]. This is an important aspect because free radicals generated from ischemic placenta, characteristic of the early stage of preeclampsia (stage 1), have been considered to play an essential role in the pathogenesis of this disease [37].

This study had some limitations. Firstly, we screened only a single concentration (100 μg/mL) of the drugs. There are few studies on cell experiments on Kampo drugs, and there is therefore no uniform lysis method. In addition, they contain various combinations of crude drugs, and it is difficult to determine an appropriate cell experiment concentration. Therefore, concentration was determined based on a previous study [18]. Furthermore, it is worth trying a variable combination of crude drugs to create a more ideal Kampo drug that increases PlGF. Secondly, we did not conduct experiments to explore the mechanism by which PlGF increases, and it is unknown whether it has the same effect *in vivo*. In addition, it has not been confirmed whether TS could promote placental development in stage 1.Future studies using primary trophoblastic culture cells or animal models of preeclampsia to explore its mechanism are needed.

## Conclusions

We demonstrated that TS induces PlGF and has an angiogenic effect, suggesting a protective effect of TS against preeclampsia (by altering the antiangiogenic state that led to the maternal endothelial damage). Moreover, we showed that screening a library of Kampo drugs might lead to the discovery of new therapeutic agents for the treatment of diseases for which no effective treatment exists.
